# Synthesis and Characterization of High-Purity, High-Entropy Diboride Ceramic Powders by a Liquid Phase Method

**DOI:** 10.3390/ma16237431

**Published:** 2023-11-29

**Authors:** Weilu Gong, Tiyuan Wang, Wei Luo, Youpei Du, Li Ye, Riheng Song, Haifeng Cui, Tong Zhao, Wei Yang, Zhen Dai, Yiqiang Hong

**Affiliations:** 1 Key Laboratory of Science and Technology on High-Tech Polymer Materials, Institute of Chemistry, Chinese Academy of Sciences, Beijing 100190, China; weilugong@iccas.ac.cn (W.G.); m15735167760@163.com (R.S.); tzhao@iccas.ac.cn (T.Z.); 2 University of Chinese Academy of Sciences, Beijing 100049, China; 3 Beijing System Design Institute of Mechanical-Electrical Engineering, Beijing 100871, Chinasq_luowei@buaa.edu.cn (W.L.); daizhen@iccas.ac.cn (Z.D.)

**Keywords:** liquid-phase precursor method, high-entropy diboride powders, solid solutions, microstructure, pyrolysis mechanism

## Abstract

A nano-dual-phase powder with ultra-fine grain size was synthesized by the liquid precursor method at 1200 °C. A series of single-phase high-entropy ceramic powders ((Ti, Zr, Hf, Nb)B_2_, (Ti, Zr, Hf, Nb, Ta)B_2_, (Ti, Zr, Hf, Nb, Mo)B_2_, (Ti, Zr, Hf, Nb, Ta, Mo)B_2_) with high purity (C content less than 0.9 wt% and O content less than 0.7 wt%) and ultrafine (average grain sizes of 340–570 nm) were successfully synthesized at 1800 °C. The sample of (TiZrHfNbTa)B_2_ exhibited a hexagonal close-packed (HCP) structure, and the metal elements were uniformly distributed at the nanoscale, microscale, and macroscale. This method did not apply to the preparation of all high-entropy ceramic powders and was unfavorable for the formation of single-phase high-entropy borides when the size difference factor exceeded 3.9%. The present work provides a guide for the development of ceramic-based composites through precursor impregnation pyrolysis.

## 1. Introduction

High-entropy transition metal diborides belonging to the hexagonal crystal system are a polymeric solid solution with an AlB_2_ structure [1], where the metal atom layer and boron layer are arranged alternately in the c-axis direction and the transition metal atoms are randomly arranged in the metal layer [2]. Meanwhile, boron atoms are bonded by covalent bonds and a mixture of ionic and covalent bonds between metal and boron atoms [3]. The excellent properties, such as high melting point (>3000 K), high hardness, good chemical stability, and high temperature stability [4,5,6,7], attribute to the strong force between the chemical bonds. Therefore, it has good application prospects in aerospace, cutting tools, microelectronics, and nuclear reactors [8,9,10].

Yan Zhang et al. [11] synthesized a high-entropy ceramic (Hf, Zr, Ta, Cr, Ti)B_2_ with high hardness (more than 29 GPa) and high toughness (more than 4.63 MPa m^1/2^) due to over 99% densification. In 2023, Steven M. Smith II et al. [12] obtained (Cr, Hf, Ta, Ti, Zr)B_2_ and (Hf, Ta, Ti, V, Zr)B_2_ ceramics for the first time with densities close to 100% by pressureless sintering. Secondary phases were present in (Hf, Ta, Ti, W, Zr)B_2_ and (Hf, Mo, Ti, W, Zr)B_2_ ceramics, which did not attain full density. Generally, the HEB materials were difficult to densify due to a low self-diffusion rate and the presence of impurities [13]. Therefore, the synthesis of high-entropy metal diboride powders is essential for excellent-performance ceramics, with current research predominantly focused on transition metals from groups IVB, VB, and VIB [14,15]. In 2016, Joshua Gild et al. [16] made the pioneering effort to prepare seven quinary boride ceramic bulk materials, including (Hf, Zr, Ta, Nb, Ti)B_2_, (Hf, Zr, Ta, Mo, Ti)B_2_, (Hf, Zr, Mo, Nb, Ti)B_2_, (Hf, Mo, Ta, Nb, Ti)B_2_, (Mo, Zr, Ta, Nb, Ti)B_2_, (Hf, Zr, W, Mo, Ti)B_2_, and (Hf, Zr, Ta, Cr, Ti)B_2_. XRD results show that all systems, except (Mo, Zr, Ta, Nb, Ti)B_2_, form a single solid solution of ceramic. However, the majority of the systems have varied degrees of elemental inhomogeneity, according to the EDS examination of ceramics. This is primarily due to the ultra-high melting point and strong covalent bonding characteristics of borides, which make it challenging for them to form uniform solid solutions. In 2019, Tallarita G et al. [17] mixed equimolar amounts of Ti, Hf, Nb, Ta, and Mo metal powders with amorphous boron powder and used self-propagating high-temperature synthesis (SHS) to prepare (Hf, Mo, Ta, Nb, Ti)B_2_ ceramic powders. However, the powders obtained by this method exhibited low sintering density, poor mechanical properties, and a noticeable enrichment of Ti elements. Wei-Min Guo et al. synthesized (Hf, Zr, Ta, Cr, Ti)B_2_, (Hf, Mo, Zr, Nb, Ti)B_2_, and (Hf, Mo, Ta, Nb, Ti)B_2_ boride high-entropy ceramic powders using metal oxide and amorphous boron powders as raw materials by boron-thermal reduction at 1600 °C for 24 h [18]. However, XRD characterization results showed the presence of certain oxide impurities, including m-(Zr, Hf)O_2_ and t-ZrO_2_, in the powders. Hence, there is an urgent need to synthesize high-purity, high-entropy metal diboride powders to further promote the development of high-entropy boride ceramics. Yan Zhang et al. [11], using B, ZrO_2_, HfO_2_, Ta_2_O_5_, Nb_2_O_5_, and TiO_2_ powders as raw materials, produced a nano-dual-phase powder by molten salt-assisted borothermal reductions at 1100 ◦C. The as-synthesised powder has a specific surface area of 22.971 m^2^/g, according to the BET measurement result, implying good sinterability. Dong Zhijun et al. [19] used precursor polyboron nitride (PBN) as the boron source and an organic precursor of HfC to synthesize HfB_2_ ceramic powders with particle sizes of about 200 nm through a liquid-phase route at 1500 °C. (Hf, Nb, Cr, Ta, Mo)B_2_ ceramic powder with an average particle size of 62.09 nm was obtained by the sol-gel method at a relatively low temperature of 1650 °C. Molten salt and sol-gel methods contribute to the synthesis of low-temperature fine grains, but the processability of these methods can be inferior. The liquid-phase precursor method offers distinct advantages in producing high-purity, ultrafine, and uniformly distributed ceramic powders [20,21]. Meanwhile, the method exhibits good process performance, which contributes to the formation of high-performance ceramic fibers and ceramic composites [22,23].

In this study, we utilized the liquid-phase precursor method to synthesize a series of high-purity, high-entropy metal diboride powders. The relationship between the size difference factor of the system and the physical phase structure of the product is also investigated. The effect of the ratio of boron to carbon to metal sources on the purity and phase structure of the product was investigated using (Ti, Zr, Hf, Nb, Ta)B_2_. We then delved deeper into the mechanism of precursor decomposition, providing the morphology, microstructure, and compositional homogeneity of the powder, as well as the oxygen content.

## 2. Experimental

### 2.1. Materials and Preparation

**Figure 1:**
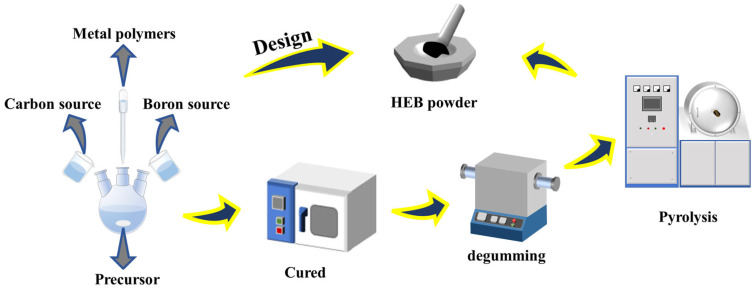
Schematic diagram of the synthesis procedure for liquid polymer and ceramic powders.

The general flow chart for synthesizing high-entropy boride (HEB) powder is shown in Figure 1. In this work, metal alkoxides (MA, M = Ti, Zr, Hf, Nb, Ta, Mo, W) were used as the initial metal sources. TiA (titanium n-propoxide, Ti(OC_3_H_7_)_4_, purity > 99 wt%, in n-propanol) and ZrA (zirconium n-propoxide, Zr(OC_3_H_7_)_4_, purity > 72 wt%, in n-propanol) were purchased from Heruidong Co., Ltd., Shandong, China. HfA, NbA, WA, MoA, and TaA were prepared by the corresponding metal chloride; for instance, TaA was synthesized by the reaction of TaCl_5_ and n-propanol, using triethylamine (Et_3_N) as a precipitant with a TaCl_5_/ n-propanol /Et_3_N molar ratio of 1/5/5 and glycol dimethyl ether as a solvent. The precipitate was removed by filtration to obtain a clear solution. Acetylacetone (Hacac) was added to MAs at room temperature with a mole ratio of MA:Hacac = 1:1 to obtain stable solutions of metal acetylacetonate alkoxides (MAAs). Afterwards, controlled co-hydrolysis and polycondensation reactions were achieved by the dropwise addition of deionized water with a molar ratio of MAAs: H_2_O = 1:1, followed by distillation to obtain metal polymers. Borate ester (BE, self-made in our laboratory) and allyl-functional novolac (AN) resin (self-made in our laboratory) acted as sources of boron and carbon, which were respectively dissolved in normal propyl alcohol and added to the solution of metal polymers. The proportion of the molarities of the metal source, the molarities of the boron source, and the mass of the carbon source (M: B: C) were studied to obtain high-purity, high-entropy borides. The mixture was continually stirred at 25 °C for 2 h to obtain a homogenous solution. The solvent was partially removed to obtain the HEB precursor by rotary evaporation. Subsequently, precursors were heated at 100 °C, 140 °C, 180 °C, 200 °C, 220 °C, and 250 °C each for an hour. The obtained powders were heated in an alumina tube furnace to remove substances released at 400 °C for 2 h in an argon atmosphere. Afterwards, the above samples were pyrolyzed to undergo a boro/carbothermal reduction reaction with a heating rate of 10 °C/min at 600 °C, 800 °C, 1000 °C, 1200 °C, 1400 °C, 1600 °C, and 1800 °C for 4 h to obtain ceramic powders in a graphite furnace.

### 2.2. Characterization

Thermogravimetry Analysis (TGA, STA 449F5) was used to analyze the transformation process of polymer to ceramic. X-ray diffraction (XRD, PANalytical Empyrean, Almelo, The Netherlands) was carried out to investigate the crystal structure of HEB powders with Cu Kα radiation at 40 KV and 40 mA. The diffraction patterns were scanned from 10° to 90° of 2θ in a step-scan mode at a step of 0.026° and a scanning speed of 4°/min. Scanning electron microscopy (SEM, SU8020, Hitachi Limited, Tokyo, Japan) and high-resolution transmission electron microscopy (HR-TEM, JEOL JEM2100 F, Tokyo, Japan), both outfitted with energy dispersive spectroscopy (EDS), were used to examine the morphologies of HEB powders. The oxygen and carbon content of the HEB powders were respectively determined by an Oxygen /Nitrogen Analyzer (TC-600C, Leco, St. Joseph, MO, USA) and a Carbon/Sulphur Analyzer (CS844, Leco, St. Joseph, MO, USA). The contents of boron and other metal elements in the powders were measured Inductively Coupled Plasma Optical Emission Spectrometer (ICP-OES, IRIS Intrepid II, Thermal, Acton, MA, USA). The software Jade 6.5 was used to calculate the lattice parameter and crystal plane spacing.

## 3. Results and Discussion

### 3.1. Factor Analysis of Size Difference

HEBs are a type of multi-component solid solution with a single-phase structure. The lattice parameter of the transition metal boride has a great influence on the formation of single-phase solid solutions. Therefore, before conducting the synthesis experiments, we calculated the size difference factor δ to assess the structural compatibility of the boride components [24]. A smaller δ value indicates a closer structural match, while a larger value suggests greater divergence. The formula for calculating δ in high-entropy borides is as follows [24]:

(1) $$\delta =\sqrt{\sum_{i=1}^{n}\frac{n_{i}}{2}\left[{\left(1-\frac{a_{i}}{\bar{a}}\right)}^{2}+{\left(1-\frac{c_{i}}{\bar{c}}\right)}^{2}\right]}$$

In this equation, n represents the total number of components constituting the high-entropy boride, $n_{i}$ is the molar fraction of the MeB_2_ component for each constituent, and *ai* and *ci* are the numerical values of the lattice parameters a and c for the corresponding hexagonal boride. $\bar{a}$ and $\bar{c}$ denote the average values of a and c, respectively, for the binary borides comprising the high-entropy material. Typically, a smaller size difference factor δ implies a closer structural resemblance among the components, facilitating the formation of high-entropy materials. We calculated the δ values for the high-entropy transition metal borides to be synthesized in this experiment based on reported boride lattice parameter values, and the results are presented in Table 1. Table 1 reveals that the size difference factors for various 4–6 component high-entropy borides fall within the range of 2.811% to 4.168%. The lattice parameters of (Ti, Zr, Hf, Ta, W)B_2_ and (Ti, Zr, Hf, Mo, W)B_2_ are the average values of the lattice parameters of the five binary borides, and these specific values are listed in Table S1.

**Figure 2:**
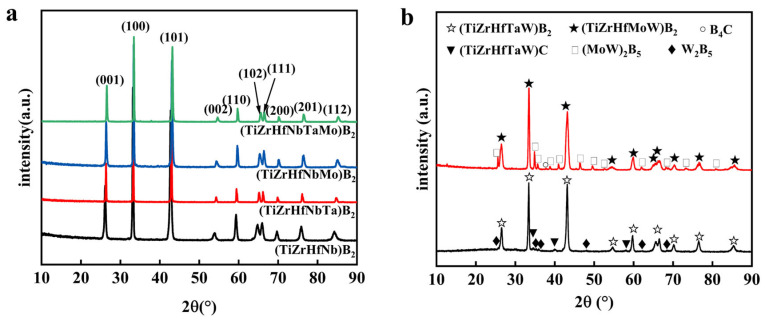
XRD patterns of the as-synthesized powders: (**a**) (Ti, Zr, Hf, Nb)B_2_, (Ti, Zr, Hf, Nb, Ta)B_2_, (Ti, Zr, Hf, Nb, Mo)B_2_, and (Ti, Zr, Hf, Nb, Ta, Mo)B_2_. (**b**) (Ti, Zr, Hf, Ta, W)B_2_ and (Ti, Zr, Hf, Mo, W)B_2_.

To investigate the correlation between the size difference factor (δ) and the phase composition of the samples, we performed XRD analysis on six different high-entropy borides. The results are presented in Figure 2. From the graph, it is evident that (Ti, Zr, Hf, Nb)B_2_, (Ti, Zr, Hf, Nb, Ta)B_2_, (Ti, Zr, Hf, Nb, Mo)B_2_, and (Ti, Zr, Hf, Nb, Ta, Mo)B_2_ all exhibit a single-phase structure, with their respective lattice parameters listed in Table 1. In contrast, (Ti, Zr, Hf, Ta, W)B_2_ and (Ti, Zr, Hf, Mo, W)B_2_ show variations. In the XRD patterns of (Ti, Zr, Hf, Ta, W)B_2_ and (Ti, Zr, Hf, Mo, W)B_2_ systems, in addition to the high-entropy phase, impurity phases such as (Mo, W)_2_B_5_, W_2_B_5_, and minor amounts of (Ti, Zr, Hf, Mo, W)C and B_4_C were observed. Notably, these non-single-phase (Ti, Zr, Hf, Ta, W)B_2_ and (Ti, Zr, Hf, Mo, W)B_2_ systems corresponded to higher δ values. Hence, it is less likely to form single-phase, high-entropy borides when δ exceeds 3.9%. In the upcoming experiments, our focus will be on (Ti, Zr, Hf, Nb, Ta)B_2_, which boasts the smallest δ value.

**Figure 3:**
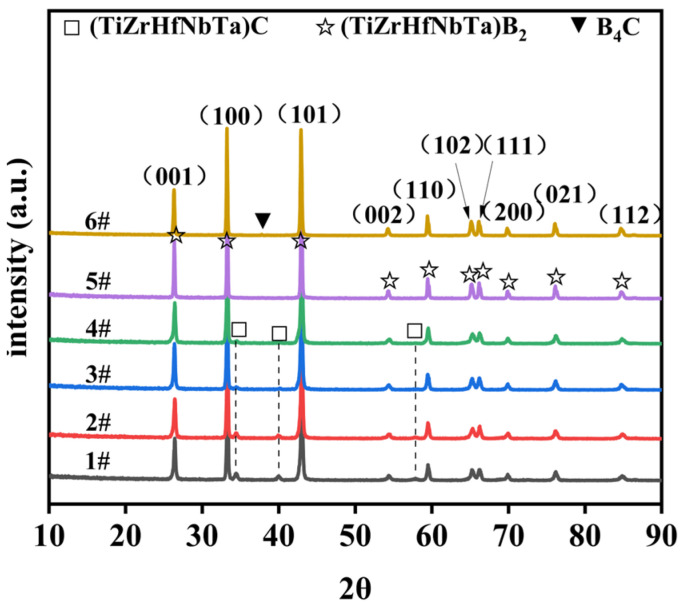
XRD patterns of the different proportions of (Ti, Zr, Hf, Nb, Ta)B_2_ powders. 1#–6# represent ceramic powders pyrolyzed at 1800 with different contents of boron and carbon sources, and these specific proportions can be found in Table 2.

In the process of preparing high-entropy borides through the liquid precursor method, the quantities of boron and carbon sources play a vital role in determining the phase structure and purity of the final product. Therefore, the amounts of boron and carbon sources are investigated to find the optimal synthesis recipe. The specific ratios of the raw materials are listed in Table 2. This table includes metal-to-boron molar ratios of 1:8, 1:10, and 1:16, as well as metal-to-carbon mass ratios of 1:30 and 1:40. To examine the phase structure of the final products, XRD tests were performed on products synthesized with different ratios. Figure 3 reveals that the optimal composition, yielding a single-phase boride structure, is achieved with a metal-to-boron-to-carbon ratio of 1:16:30. When the metal-to-boron ratio falls below 1:16, it results in the presence of corresponding metal carbide phases. When the ratio of metal and carbon is greater than 1:30 and the boron source is moderate, the impurity phase of B_4_C is detected. The elemental contents of the as-synthesized powder at the optimum ratio are listed in Table 3, illustrating that the proportions of metal elements in the powder are nearly 1:1:1:1:1, and the metal-to-boron ratio is close to 1:2, with lower contents of carbon and oxygen. Excessive boron and oxygen elements indicate that boron oxide impurities are likely to be present in the sample. Hence, 1:16:30 (M: B: C) represents the best synthesis proportion, and exploring the quantities of boron and carbon sources is of great significance.

### 3.2. Thermogravimetric Analysis of Precursors

**Figure 4:**
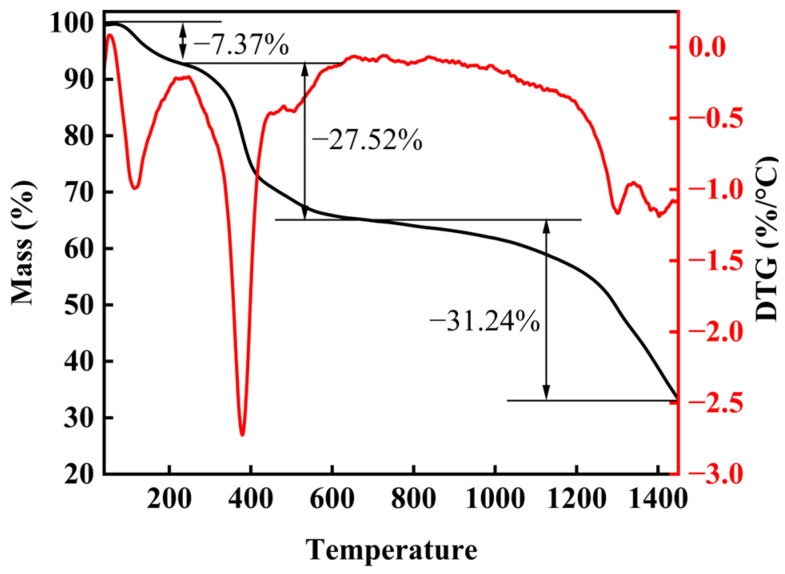
TG-DTG describes the pyrolysis process of the (Ti, Zr, Hf, Nb, Ta)B_2_ curing sample.

To gain a deeper insight into the pyrolysis process of high-entropy boride (HEB) precursors, we conducted a study on the pyrolysis process of the cured products using thermogravimetric analysis (TG). As depicted in Figure 4, the TG-DTG curves reveal a pyrolysis process comprising three distinct phases. The first part, extending from room temperature to 237 °C, is associated with a weight loss of approximately 7.37 wt%. This initial weight loss is primarily attributed to the removal of hydroxyl groups present in the sample and a small amount of adsorbed water vapor. The second part occurs within the temperature range of 237 °C to 680 °C and results in a weight loss of about 27.52 wt%. This phase signifies the transformation from organic to inorganic materials, involving the breakdown of organic-inorganic molecular chains and the substantial departure of organic groups. The final phase is linked to the boron-carbon thermal reduction process, potentially leading to the release of CO, CO_2_, and B_2_O_3_ [26]. This phase is marked by a substantial weight reduction of 31.24 wt%. During this stage, metals gradually form borides, laying the groundwork for the ultimate formation of a single-phase high-entropy boride. These discoveries hold significant importance for comprehending and refining the preparation process for high-entropy borides.

### 3.3. Analysis of the Pyrolysis Process of Precursors

**Figure 5:**
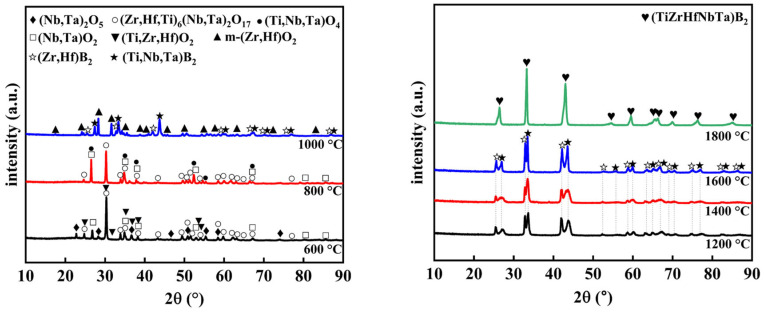
XRD patterns of (Ti, Zr, Hf, Nb, Ta)B_2_ powders obtained at 600–1800 °C.

We conducted further research into the transformation of the (Ti, Zr, Hf, Nb, Ta)B_2_ precursor from a polymer to ceramics using X-ray diffraction (XRD). Figure 5 illustrates the results of this investigation, showing the evolution of phases at different heat treatment temperatures. Samples treated at 600 °C are predominantly composed of oxide phases, including (Nb, Ta)_2_O_5_, (Zr, Hf, Ti)_6_(Nb, Ta)_2_O_17_, (Ti, Zr, Hf)O_2_, and (Nb, Ta)O_2_. As the annealing temperature increases to 800 °C, (Nb, Ta)_2_O_5_ and (Ti, Zr, Hf)O_2_ gradually transform into (Ti, Nb, Ta)O_4_. When the temperature is further raised to 1000 °C, oxide phases such as m-(Zr, Hf)O_2_ undergo a reaction with B and C elements, resulting in a two-phase structure primarily consisting of (Zr, Hf)B_2_ and (Ti, Nb, Ta)B_2_. It is noteworthy that the diffraction peaks of (Zr, Hf)B_2_ are observed to the left of the diffraction peaks of (Ti, Nb, Ta)B_2_ due to the larger atomic radii of Zr and Hf. Simultaneously, the diffraction peak intensity of (Zr, Hf)B_2_ is lower than that of (Ti, Nb, Ta)B_2_, indicating a relatively lower content of (Zr, Hf)B_2_ due to the continued presence of some Zr and Hf elements in oxide form. As the heat treatment progresses from 1200 °C to 1600 °C, the powder maintains a two-phase structure, and the diffraction peaks intensity of (Zr, Hf)B_2_ gradually approaches that of (Ti, Nb, Ta)B_2_. This suggests the nearly complete transformation of metal oxides into borides. Figure 4 shows that there is still a mass loss after 1200 °C, primarily due to TG being conducted in an Ar atmosphere, which slows down the volatilization of excess boron oxide. The pyrolysis process, carried out in a vacuum, has almost entirely volatilized the boron oxide by 1200 °C. Ultimately, through element diffusion, (Zr, Hf)B_2_ and (Ti, Nb, Ta)B_2_ form a single-phase solid solution, (Ti, Zr, Hf, Nb, Ta)B_2_, in the heated samples at 1800 °C.

### 3.4. Micro/Nanostructure of (Ti, Zr, Hf, Nb, Ta)B_2_ Powders

**Figure 6:**
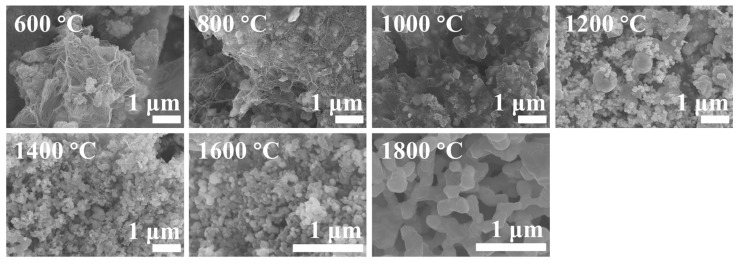
The SEM image (Ti, Zr, Hf, Nb, Ta)B_2_ powers obtained at different temperatures.

**Figure 7:**
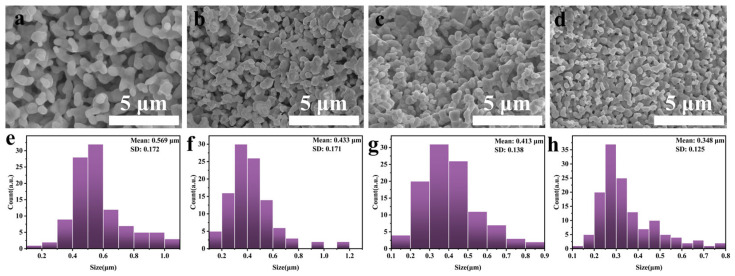
The image of the SEM and particle size distribution of ceramic powder obtained at 1800 °C. (**a**,**e**) (Ti, Zr, Hf, Nb)B_2_, (**b**,**f)** (Ti, Zr, Hf, Nb, Ta)B_2_, (**c**,**g**) (Ti, Zr, Hf, Nb, Mo**)**B_2_, (**d**,**h**) (Ti, Zr, Hf, Nb, Ta, Mo)B_2_.

**Figure 8:**
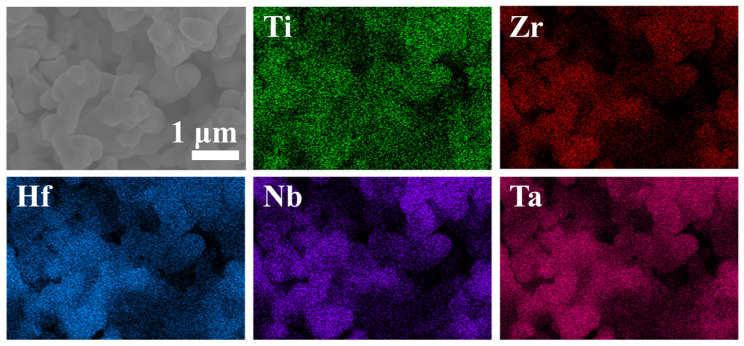
The SEM and EDS images of the (Ti, Zr, Hf, Nb, Ta)B_2_ heat-treat at 1800 °C.

As depicted in Figure 6, we conducted a scanning electron microscopy (SEM) study to examine the morphology of (Ti, Zr, Hf, Nb, Ta)B_2_ ceramics obtained at different temperatures ranging from 600 °C to 1800 °C. The morphological changes in the powders between 600 °C and 800 °C are relatively minor, primarily consisting of particles and a filament network. According to XRD results, the particles mainly consist of metallic oxides enveloped within an amorphous layer formed by the interaction of carbon and boron. The formation of filament materials is likely due to a high-temperature softening process followed by cooling and drawing, presumed to be caused by an excess of boron esters. At 1000 °C, the filament materials disappear, suggesting substantial decomposition of the boron esters at this stage. A small number of crystalline particles are enveloped within a mixture of amorphous boron oxide and carbon. According to XRD results, these particles are composed of oxides and a small amount of borides. The amorphous materials cannot be observed around the powders obtained at 1200 °C to 1400 °C. However, there are two types of particles in these powders, with the larger particles likely being the first-formed boride crystals. The powders at 1800 °C exhibit significantly increased particle size compared to those at 1600 °C, accompanied by some degree of sintering. Figure 7 demonstrates the image of SEM and particle size distributions of (Ti, Zr, Hf, Nb)B_2_, (Ti, Zr, Hf, Nb, Ta)B_2_, (Ti, Zr, Hf, Nb, Mo)B_2_, and (Ti, Zr, Hf, Nb, Ta, Mo)B_2_, which show that the ceramic samples prepared by the liquid precursor method have a particle size of approximately 340–570 nm. Figure 8 is the SEM and EDS image of the (Ti, Zr, Hf, Nb, Ta)B_2_ heat treat at 1800 °C, which revealed a uniform distribution of metallic elements at the micrometer scale with no evidence of metallic element enrichment. 

**Figure 9:**
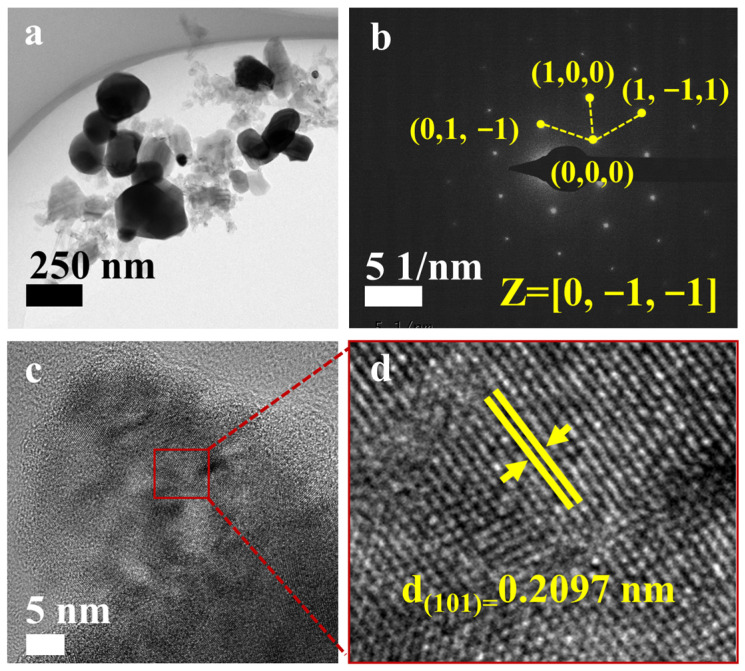
TEM analysis of the as-synthesized (Ti, Zr, Hf, Nb, Ta)B_2_ powders at 1800 °C: (**a**) TEM image; (**b**) SAED pattern; (**c**) HR-TEM micrographs; (**d**) the enlarged HR-TEM micrographs.

**Figure 10:**
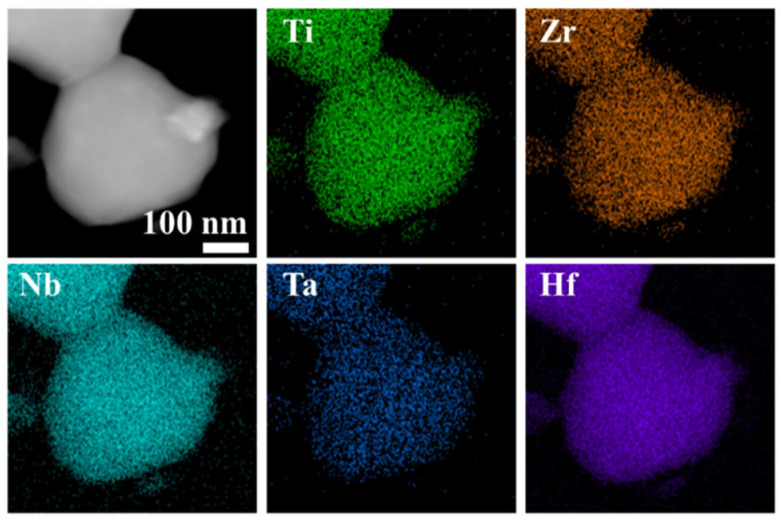
TEM image and the corresponding EDS maps of (Ti, Zr, Hf, Nb, Ta)B_2_ powders obtained at 1800 °C.

To explore the nanoscale crystal structure and compositional uniformity, we conducted further research using Transmission Electron Microscopy (TEM) on (Ti, Zr, Hf, Nb, Ta)B_2_ powder obtained at 1800 °C. Figure 9a presents a TEM image of the synthesized (Ti, Zr, Hf, Nb, Ta)B_2_ powder, revealing numerous independent nanoscale particles in the synthesized material. Dark regions indicate areas of greater sample thickness. In Figure 9b, a Selected Area Electron Diffraction (SAED) pattern is shown for the synthesized powder along the [0,−1,−1] axis. The well-organized and symmetric arrangement of diffraction spots clearly indicates that the synthesized powder possesses a single-crystal hexagonal structure. High-resolution TEM (HR-TEM) images in Figure 9c,d demonstrate the periodic lattice structure of the synthesized (Ti, Zr, Hf, Nb, Ta)B_2_ powder, with a lattice spacing of 0.2097 nm for the (101) crystal plane, closely matching the calculated value (2.1038 Å) from the XRD spectrum. Further analysis through the Energy-Dispersive X-ray Spectroscopy (EDS) in Figure 10 reveals the even distribution of all metallic elements at the nanoscale, without evidence of clustering or depletion. However, a small amount of amorphous boron oxide is present around the ceramic particles, which is consistent with the ICP test results. 

### 3.5. Speculation on the Pyrolysis Mechanism of Precursors

**Figure 11:**
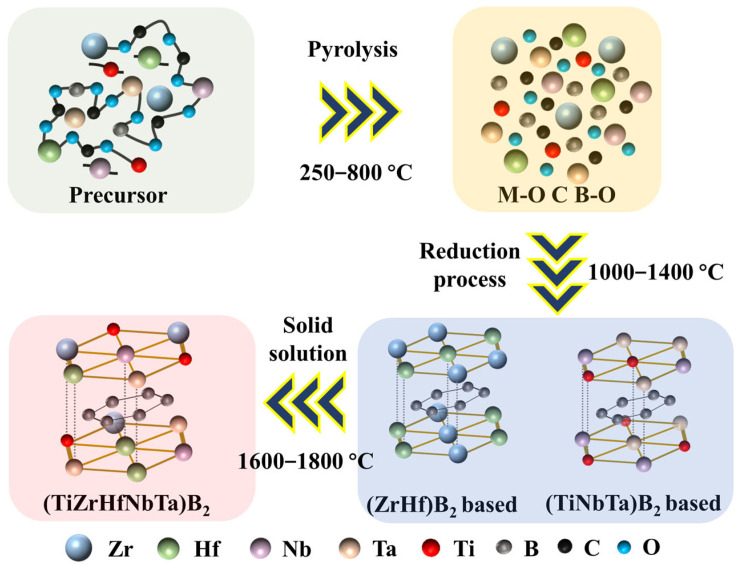
A schematic illustration showing the main process for forming single-phase (Ti, Zr, Hf, Nb, Ta)B_2_ high-entropy boron ceramic.

Based on the above analysis, we hypothesize the decomposition mechanism of the (Ti, Zr, Hf, Nb, Ta)B_2_ liquid precursor as depicted in Figure 11. During the heat treatment process below 800 °C, the liquid precursor primarily undergoes an organic-to-inorganic transformation, leading to the formation of various metal oxide solid solutions and amorphous carbon and boron oxides. When the decomposition temperature falls within the range of 1000 °C to 1400 °C, the main process involves a carbon-boron thermal reduction reaction, resulting in a two-phase structure primarily composed of (Zr, Hf)B_2_ and (Ti, Nb, Ta)B_2_. As the decomposition temperature increases from 1600 °C to 1800 °C, the process primarily entails phase solid solution, ultimately culminating in the formation of the hexagonal (Ti, Zr, Hf, Nb, Ta)B_2_ single-phase solid solution.

As a new class of ultrahigh-temperature ceramics with many attractive physicochemical properties (such as high melting point, high hardness and chemical inertness, and good electrical and thermal conductivity), high-entropy diborides can be potential candidates for high-temperature thermal protection materials in extreme environments and electrode materials. The high-purity, high-entropy boride powders in this work can be processed to obtain ceramic blocks and coating materials, which are expected to be applied to parts such as nose cones and wings of aircraft. As shown in Figure S1, we prepared high-entropy boride blocks by hot pressing and ceramic spheres by spray drying using experimentally prepared high-entropy diboride powder. This research lays the foundation for the preparation of ceramic blocks, ceramic matrix composites, and high-temperature resistant coatings.

## 4. Conclusions

In summary, we have successfully synthesized four high-purity nanoscale powders using the liquid precursor method, all of which exhibit δ values below 3.9%. In the case of the two powders with an δ value exceeding 3.9%, a secondary phase, mainly composed of W_2_B_5_ and (W, Mo)_2_B_5_, was observed. Additionally, we conducted a detailed study of the decomposition process of the (Ti, Zr, Hf, Nb, Ta)B_2_ precursors. It was observed that the precursor had already completely formed a two-boride phase, primarily composed of (Zr, Hf)B_2_ and (Ti, Nb, Ta)B_2_, at 1200 °C. Single-phase high-entropy borides with hexagonal structure are formed by solid solution at 1800 °C, with cell parameters a = b = 0.3105 nm and c = 0.3356 nm. The samples prepared by the liquid precursor method have a particle size of approximately 340–570 nm. They exhibit a high degree of compositional homogeneity, from the nanoscale to the microscale.

## Figures and Tables

**Table 1:** δ values and unit cell parameters of six kinds of high-entropy boride.

Composition	δ (%)	Lattice Parameter (Å)
(Ti, Zr, Hf, Nb)B_2_	2.925	a = b = 0.3113, c = 0.3374
(Ti, Zr, Hf, Nb, Ta)B_2_	2.811	a = b = 0.3105, c = 0.3356
(Ti, Zr, Hf, Nb, Mo)B_2_	3.315	a = b = 0.3090, c = 0.3363
(Ti, Zr, Hf, Nb, Ta, Mo)B_2_	3.078	a = b = 0.3087, c = 0.3341
(Ti, Zr, Hf, Ta, W)B_2_	3.901	a = b = 0.3084, c = 0.3310 [25]
(Ti, Zr, Hf, Mo, W)B_2_	4.168	a = b = 0.3073, c = 0.3292 [25]

**Table 2:** Raw material composition and corresponding Phase structure of (Ti, Zr, Hf, Nb, Ta)B_2_.

Samples	M:B:C	Phase Structure
1#	1:8:30	(Ti, Zr, Hf, Nb, Ta)B_2_, (Ti, Zr, Hf, Nb, Ta)C
2#	1:8:40	(Ti, Zr, Hf, Nb, Ta)B_2_, (Ti, Zr, Hf, Nb, Ta)C
3#	1:10:30	(Ti, Zr, Hf, Nb, Ta)B_2_, (Ti, Zr, Hf, Nb, Ta)C
4#	1:10:40	(Ti, Zr, Hf, Nb, Ta)B_2_, (Ti, Zr, Hf, Nb, Ta)C
5#	1:16:30	(Ti, Zr, Hf, Nb, Ta)B_2_
6#	1:16:40	(Ti, Zr, Hf, Nb, Ta)B_2_, B_4_C

**Table 3:** Element content analysis of four kinds of powders prepared at 1800 °C.

Empirical Formula	Ti	Zr	Hf	Nb	Ta	Mo	B	C	O
(Ti_0.25_Zr_0.26_Hf_0.27_Nb_0.26_)B_2.7_C_0.09_O_0.01_	9.1	17.9	36.3	18.2	/	/	22.2	0.8	0.4
(Ti_0.2_Zr_0.21_Hf_0.22_Nb_0.21_Ta_0.21_)B_2.1_C_0.08_O_0.01_	6.6	13	26.7	13.6	26.00	/	15.7	0.7	0.7
(Ti_0.2_Zr_0.21_Hf_0.2_Nb_0.2_Mo_0.2_)B_2.1_C_0.1_O_0.01_	6.7	13.2	25.3	/	25.4	13.7	15.9	0.8	0.6
(Ti_0.17_Zr_0.17_Hf_0.17_Nb_0.17_Ta_0.17_Mo_0.18_)B_2.49_C_0.19_O_0.01_	5.8	11.3	22.1	11.5	22.3	11.9	16.4	0.9	0.7

## Data Availability

Data are contained within the article and Supplementary Materials.

## Supplementary Materials

The following supporting information can be downloaded at: https://www.mdpi.com/article/10.3390/ma16237431/s1, Figure S1: (a) the image of ceramic blocks prepared by hot pressing at 1900 °C, (b) image of ceramic spheres prepared by spray drying based on (Ti, Zr, Hf, Nb, Ta)B_2_ powders prepared in this work; Table S1: The lattice parameter of (Ti_0.2_Zr_0.2_Hf_0.2_Ta_0.2_W_0.2_)B_2_ and (Ti_0.2_Zr_0.2_Hf_0.2_Mo_0.2_W_0.2_)B_2_.
