# Supplementary material for: Synthesis and Characterization of High-Purity, High-Entropy Diboride Ceramic Powders by a Liquid Phase Method
Source: Materials (Basel). 2023 Nov 29;16(23):7431. doi: 10.3390/ma16237431 (PMC10707663; doi:10.3390/ma16237431)
Supplement: Supplementary file 1 [file materials-16-07431-s001.zip › materials-2709511-supplementary.pdf]

Support Materials

Table S1 The lattice parameter of $(Ti_{0.2}Zr_{0.2}Hf_{0.2}Ta_{0.2}W_{0.2})B_2$ and $(Ti_{0.2}Zr_{0.2}Hf_{0.2}Mo_{0.2}W_{0.2})B_2$

Sample	a (nm)	c (nm)	literature
TiB_2	0.3028	0.3228	
ZrB_2	0.3169	0.3530	
HfB_2	0.3141	0.3470	
NbB_2	0.3112	0.3274	
TaB_2	0.3076	0.3265	[25]
MoB_2	0.3005	0.3173	
WB_2	0.3023	0.3057	
$(Ti_{0.2}Zr_{0.2}Hf_{0.2}Ta_{0.2}W_{0.2})B_2$	0.3084	0.331	
$(Ti_{0.2}Zr_{0.2}Hf_{0.2}Mo_{0.2}W_{0.2})B_2$	0.3073	0.3292	

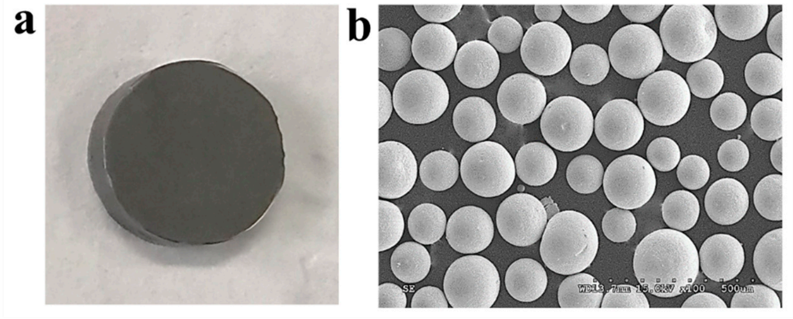

Figure S1. (a) the image of ceramic blocks prepared by hot pressing at 1900 °C, (b) image of ceramic spheres prepared by spray drying based on (Ti, Zr, Hf, Nb, Ta)B_2 powders prepared in this work.
